# Supplementary material for: The curious case of the structural phase transition in SnSe insights from neutron total scattering
Source: Nat Commun. 2023 Jun 3;14:3211. doi: 10.1038/s41467-023-38454-0 (PMC10239460; doi:10.1038/s41467-023-38454-0)
Supplement: Supplementary file 1 — Supplementary Information [file 41467_2023_38454_MOESM1_ESM.pdf]

Supplementary Information

The Curious Case of the Structural Phase Transition in SnSe Insight from Neutron Total Scattering

Bo Jiang et al.

Supplementary Figures

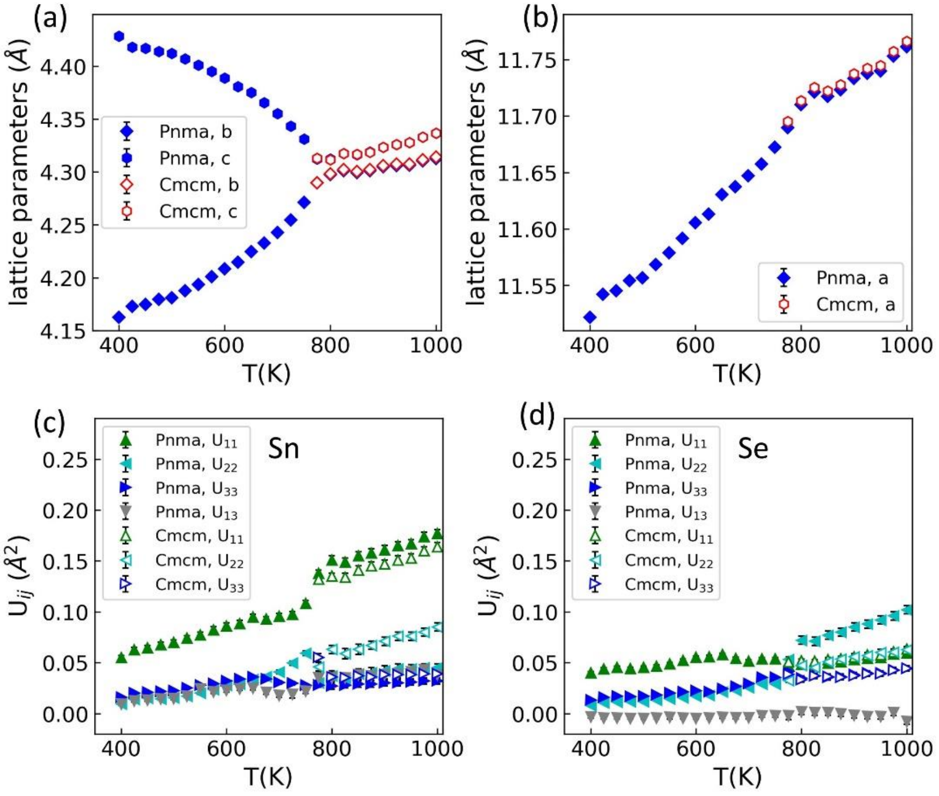

Fig. S1. Temperature dependent lattice parameters and atomic anisotropic displacement parameters (ADP) (with error bars) from Rietveld refinements using neutron diffraction data. If error bars are not visible they are smaller than the data markers.

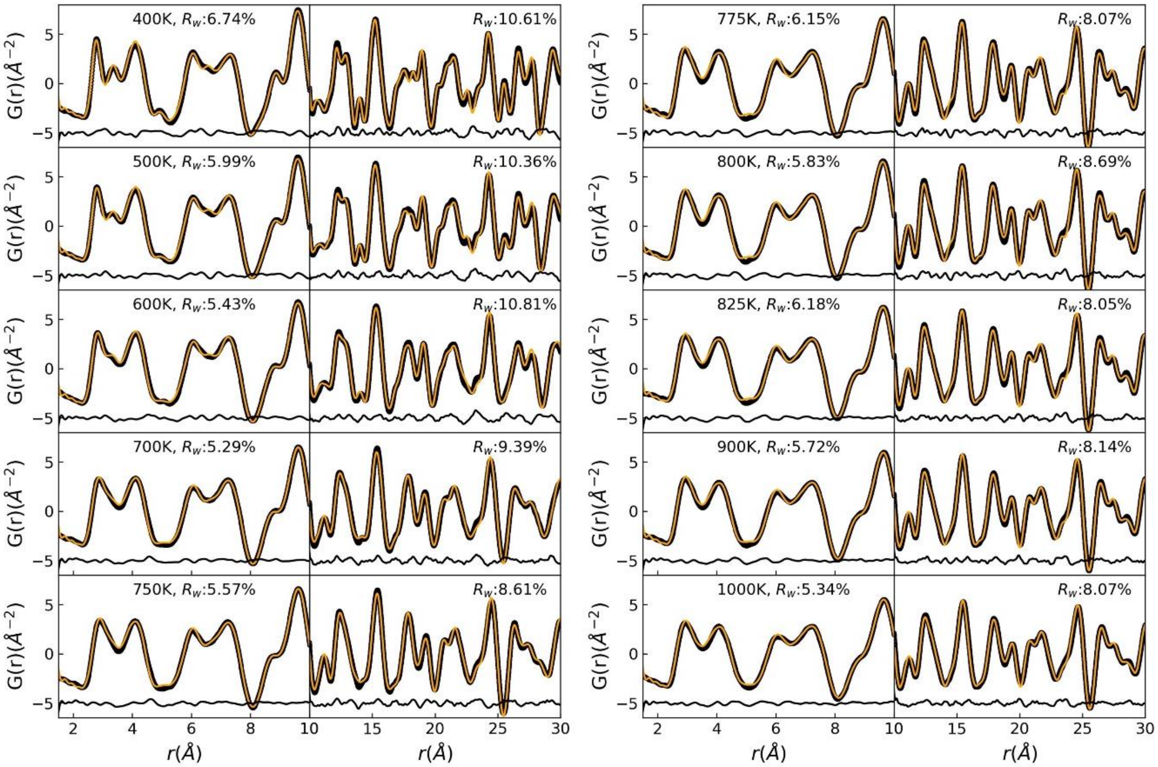

Fig. S2. Temperature dependent PDF refinements of neutron data using GeS-type (*Pnma*) space group at low-r range fits to the 1.5-10 Å PDF region and high-r range fits to the 10-30 Å. Selected temperature points are shown from 400 K to 1000 K.

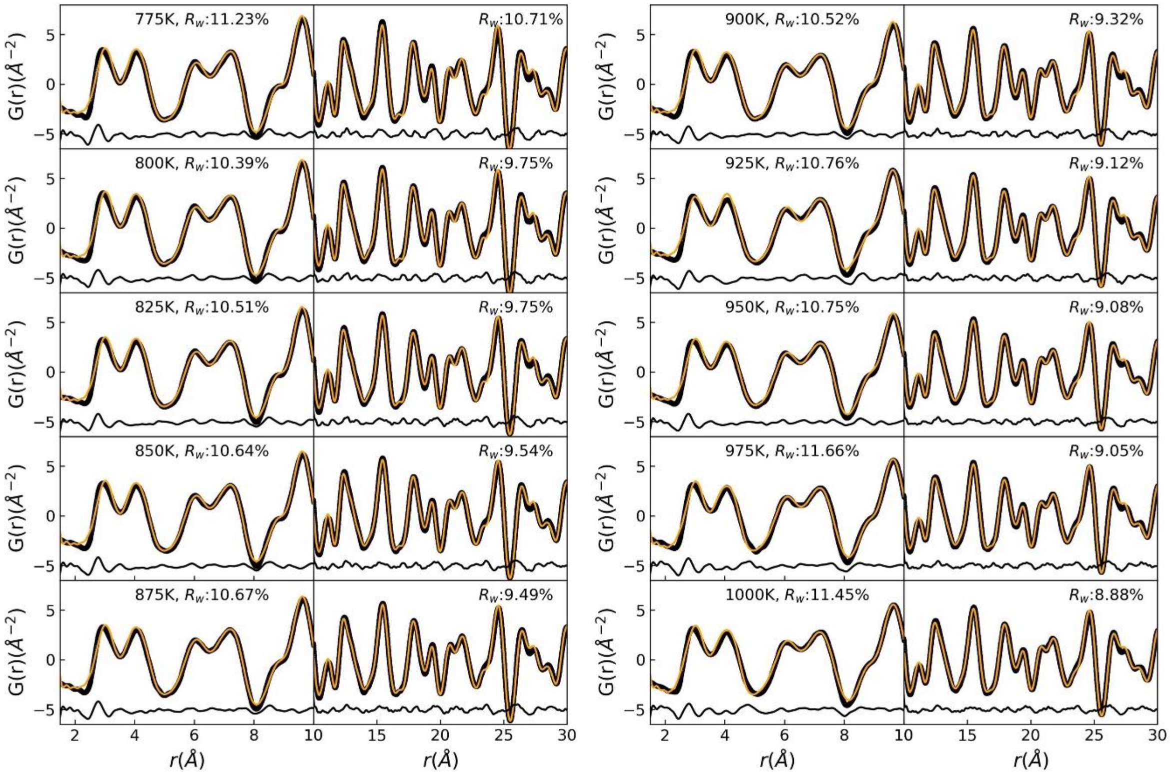

Fig. S3. Temperature dependent PDF refinements of neutron data using the TlI-type (*Cmcm*) space group model. Fits of the low-r range (1.5 Å -10 Å) PDF region are shown at a different scale relative to first of the high-r range (10 Å -30 Å) PDF region. Temperature points are shown from 775 K to 1000 K.

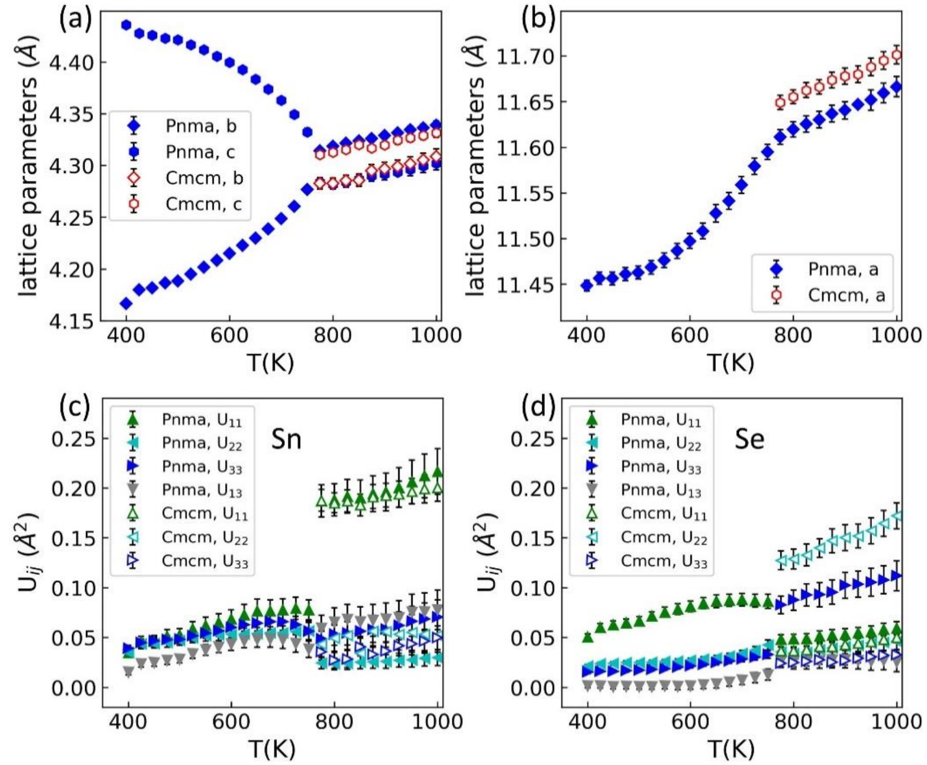

Fig. S4. Temperature dependent lattice parameters (with error bars) and atomic displacement parameters (ADP) from PDF refinements using data in the real space *r*-range of 1.5 Å to 30 Å. If error bars are not visible they are smaller than the data markers. Source data are provided as a Source Data file.

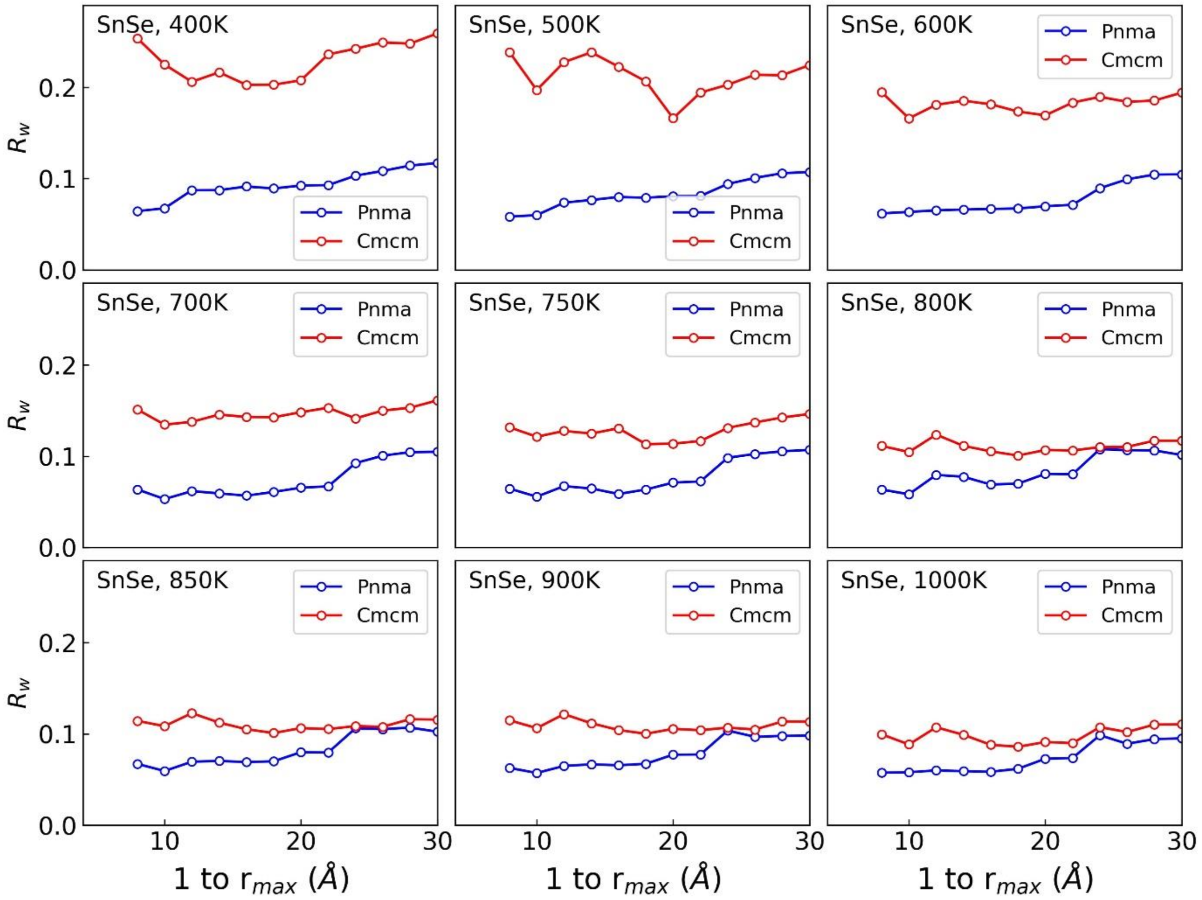

Fig. S5. A comparison of the calculated agreement factors (R_w) between refinements using room temperature phase *Pnma* and high temperature phase *Cmcm* models across varying r_{max} range of selected temperature datasets.

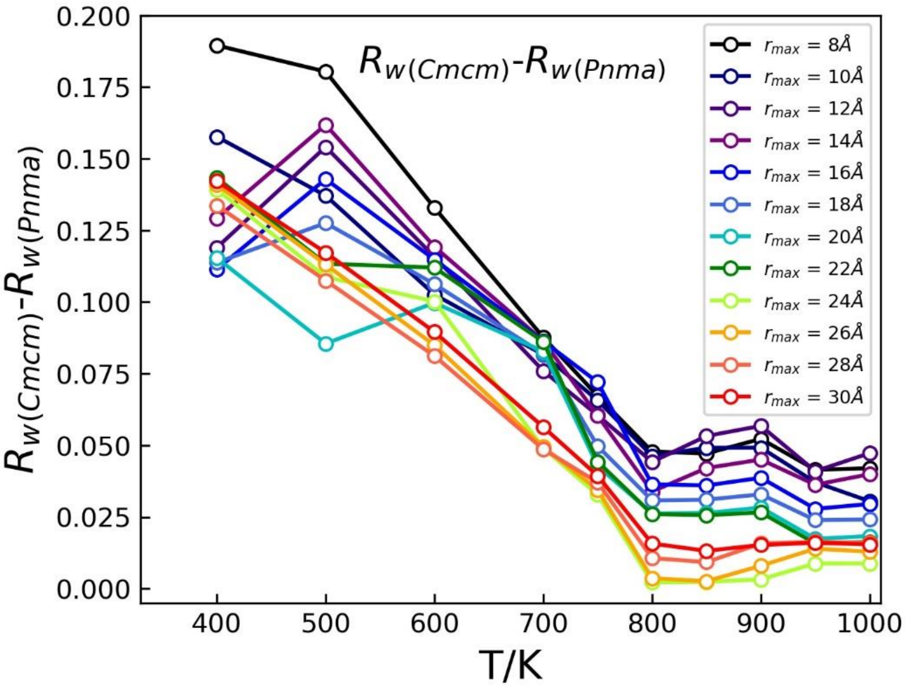

Fig. S6. The calculated agreement factor (R_w) difference between refinements using room temperature phase *Pnma* and high temperature phase *Cmcm* models across increasing r_{max} ranges of various temperature datasets. Note that the two models are symmetry related, with *Pnma* containing more refinable parameters. The fact that the *Pnma* model performs better over the entire real-space range fit (even above the average structure phase transition temperature) reflects the influence of the persistent local order observed at all temperatures.

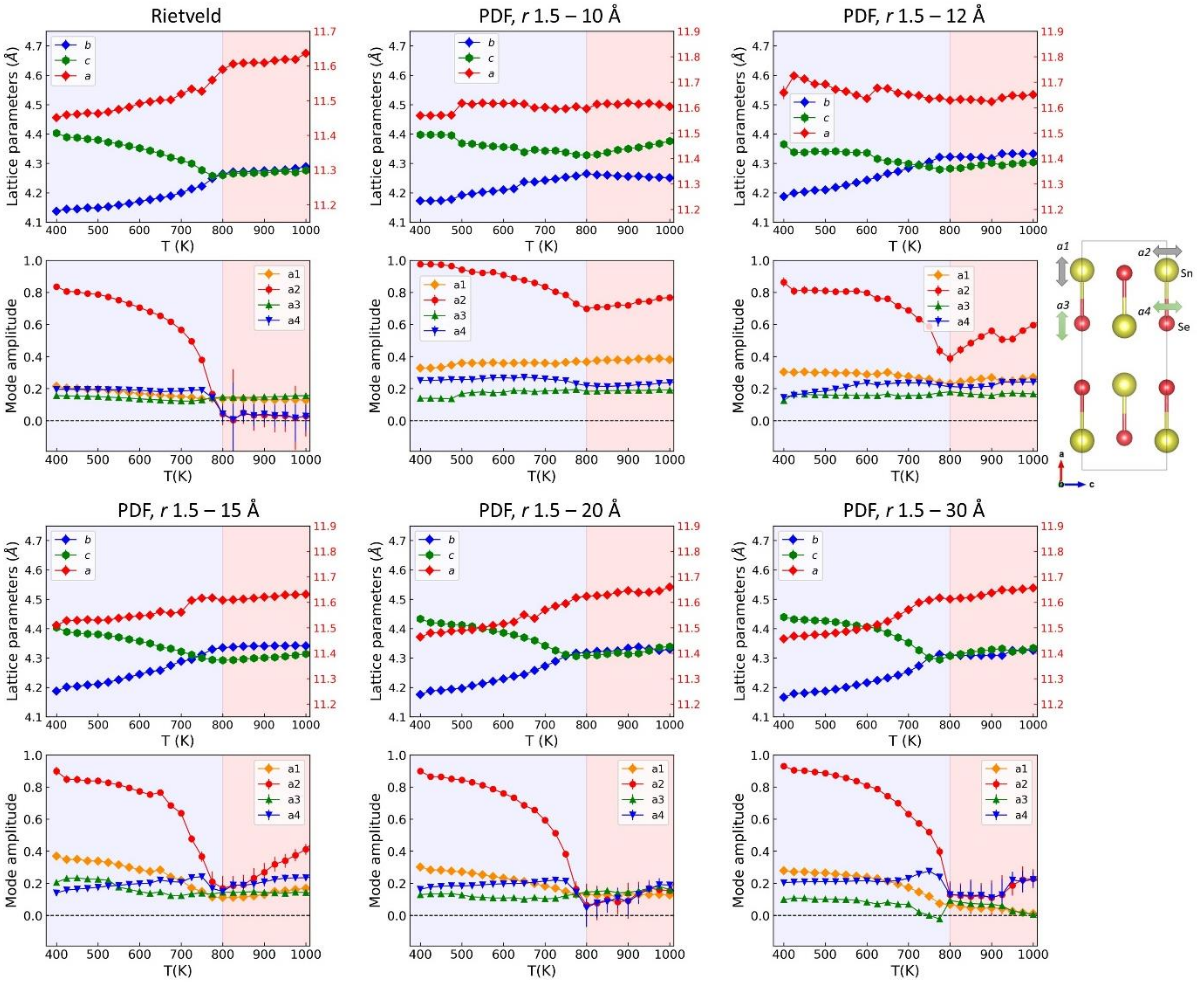

Fig. S7. Refined lattice parameters and four mode amplitudes across the SnSe phase transition, displayed separately according to discrete data length-scales. Symmetry-mode based Rietveld and PDF refinements were performed sequentially using the TOPAS v6 suite in conjunction with ISODISTORT.

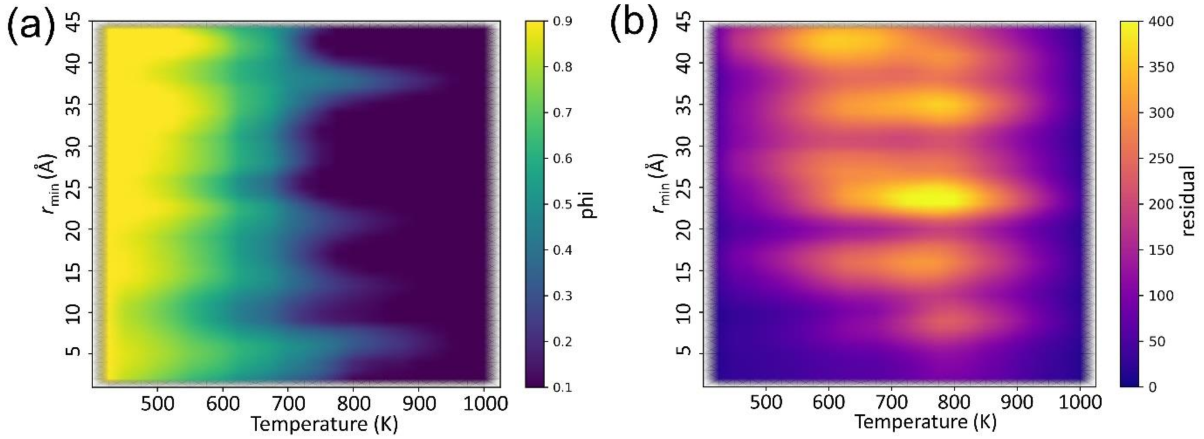

Fig. S8. (a) *r*-dependent CATS analysis performed with a constant box-width of 4 Å from ranges 1-5 Å up to 46-50 Å. (b) Residual map of qualitative difference in CATS fit quality above *r* =10 Å.

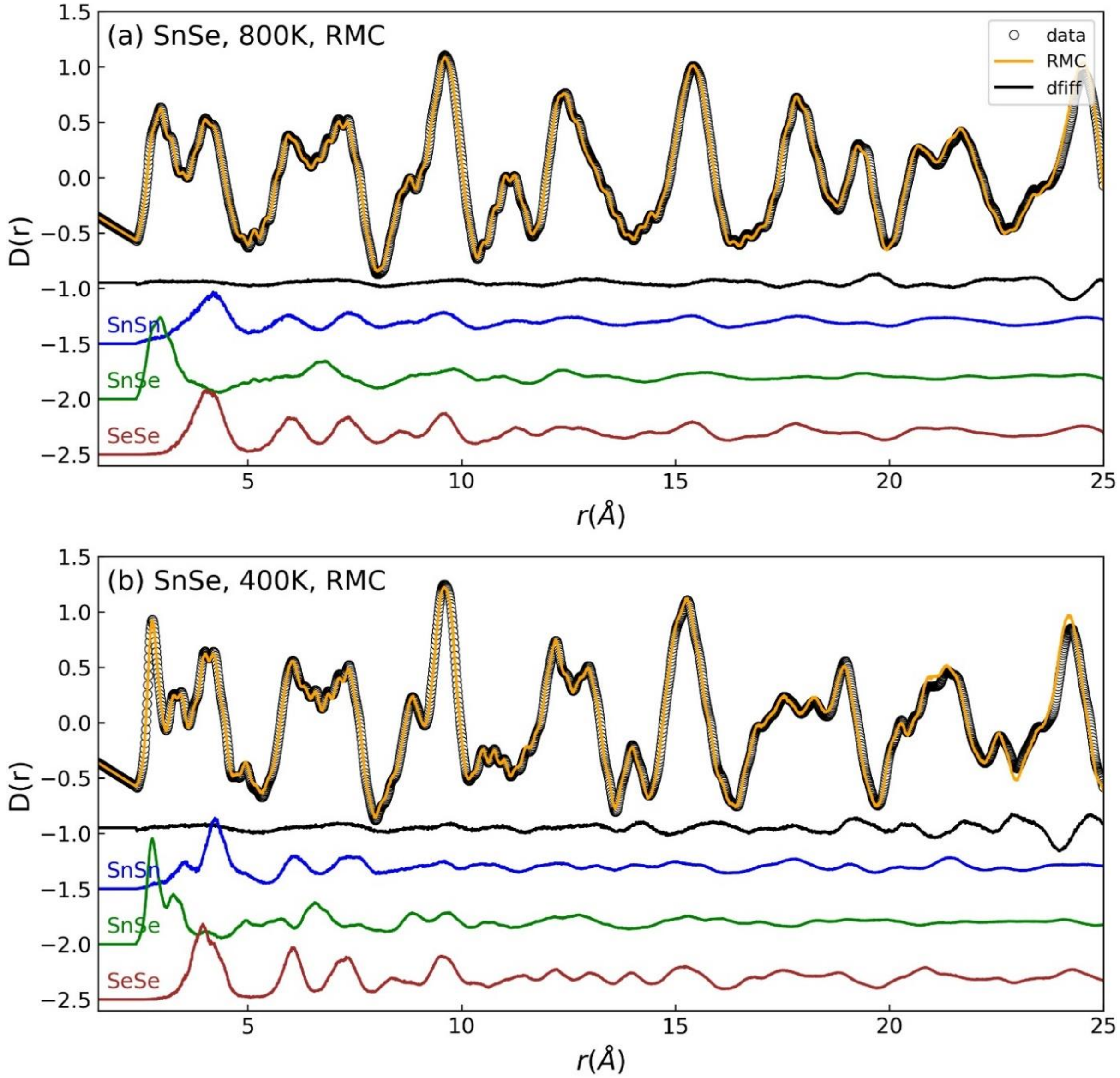

Fig. S9. Neutron total scattering fits to D(r) (black circles) in the RMC simulation (orange lines) for SnSe at (a) 800 K, and (b) 400 K, with partial pair correlation functions from models shown below as separate, labeled, colored lines. Source data are provided as a Source Data file.

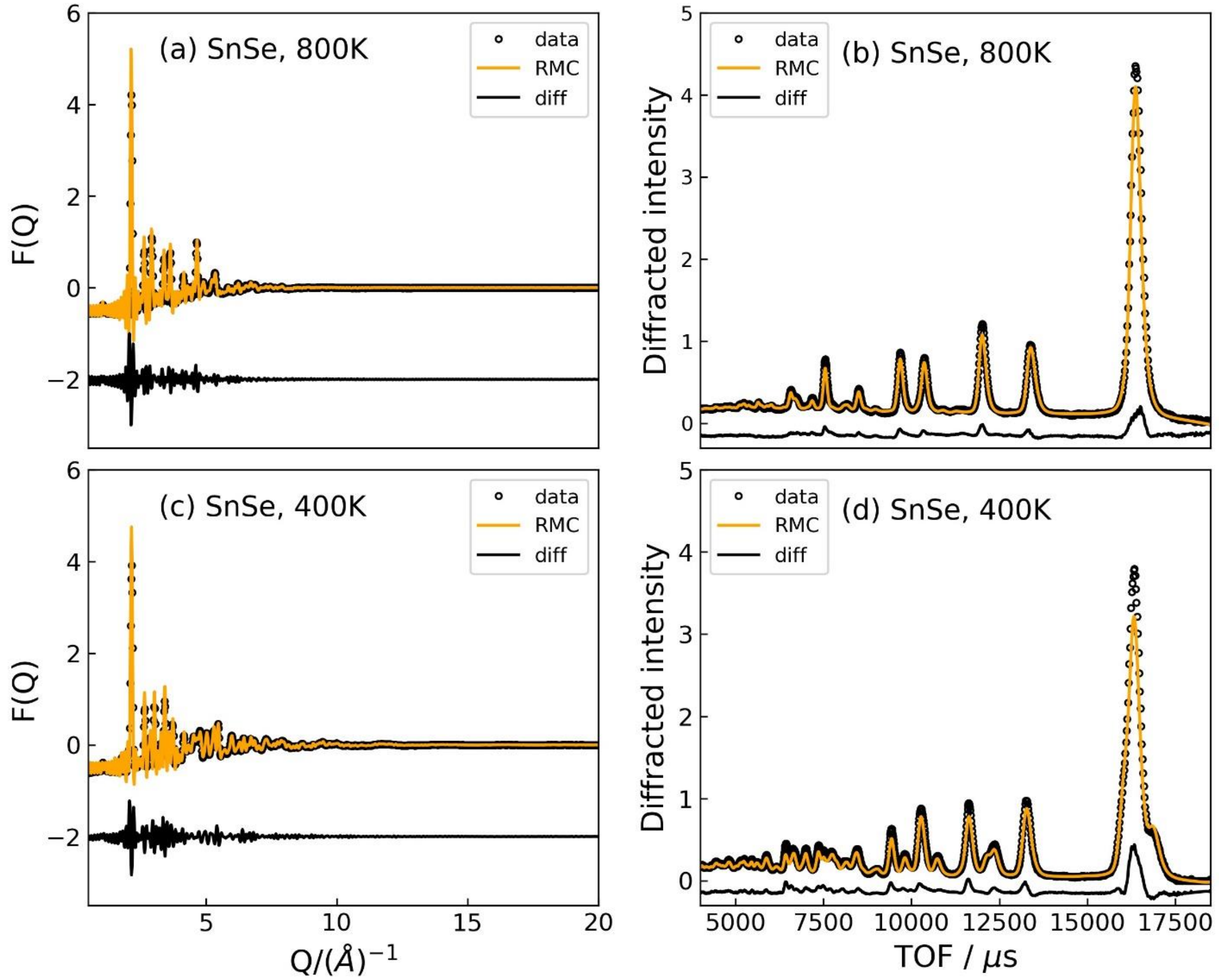

Fig. S10. Neutron total scattering fits to F(Q) and Bragg data in RMC simulation (orange lines) for SnSe at (a, b) 800 K, (c, d) 400 K. The weight assigned to D(r), F(Q) and Bragg data set were automatically adjusted during RMC simulations.

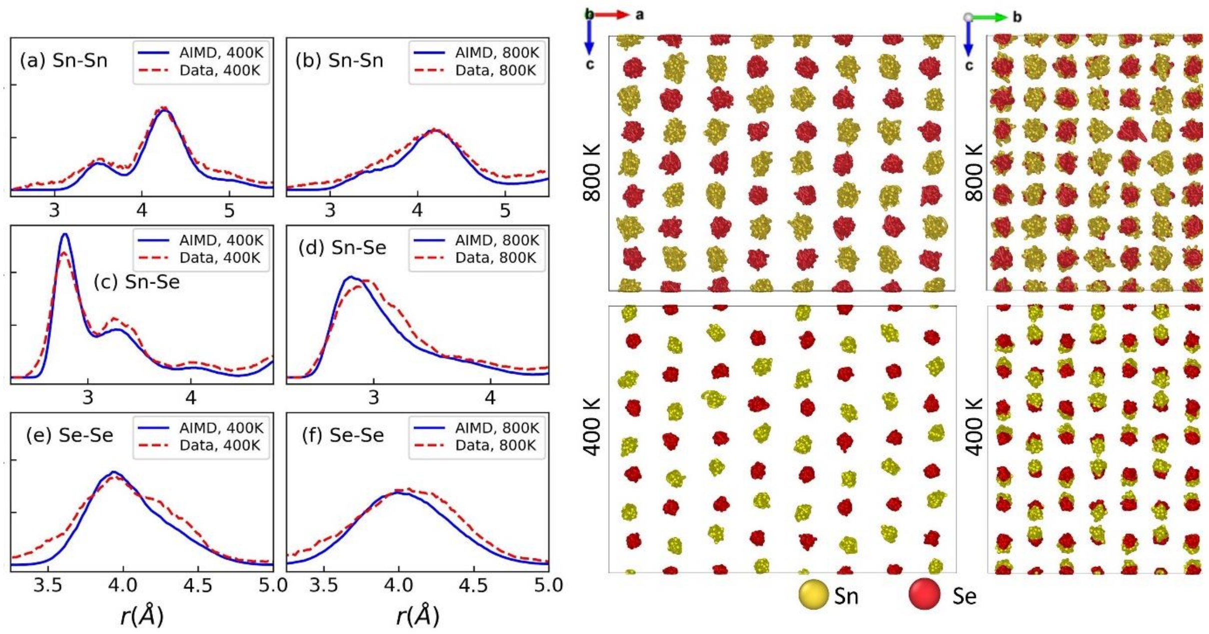

Fig. S11. Comparison of atomic pair correlations from the radial distribution function (RDF) between AIMD simulations and RMC model. Source data are provided as a Source Data file. The corresponding trajectory projections of AIMD simulations at 800 K and 400 K of SnSe derived from stable phase were shown on the right.

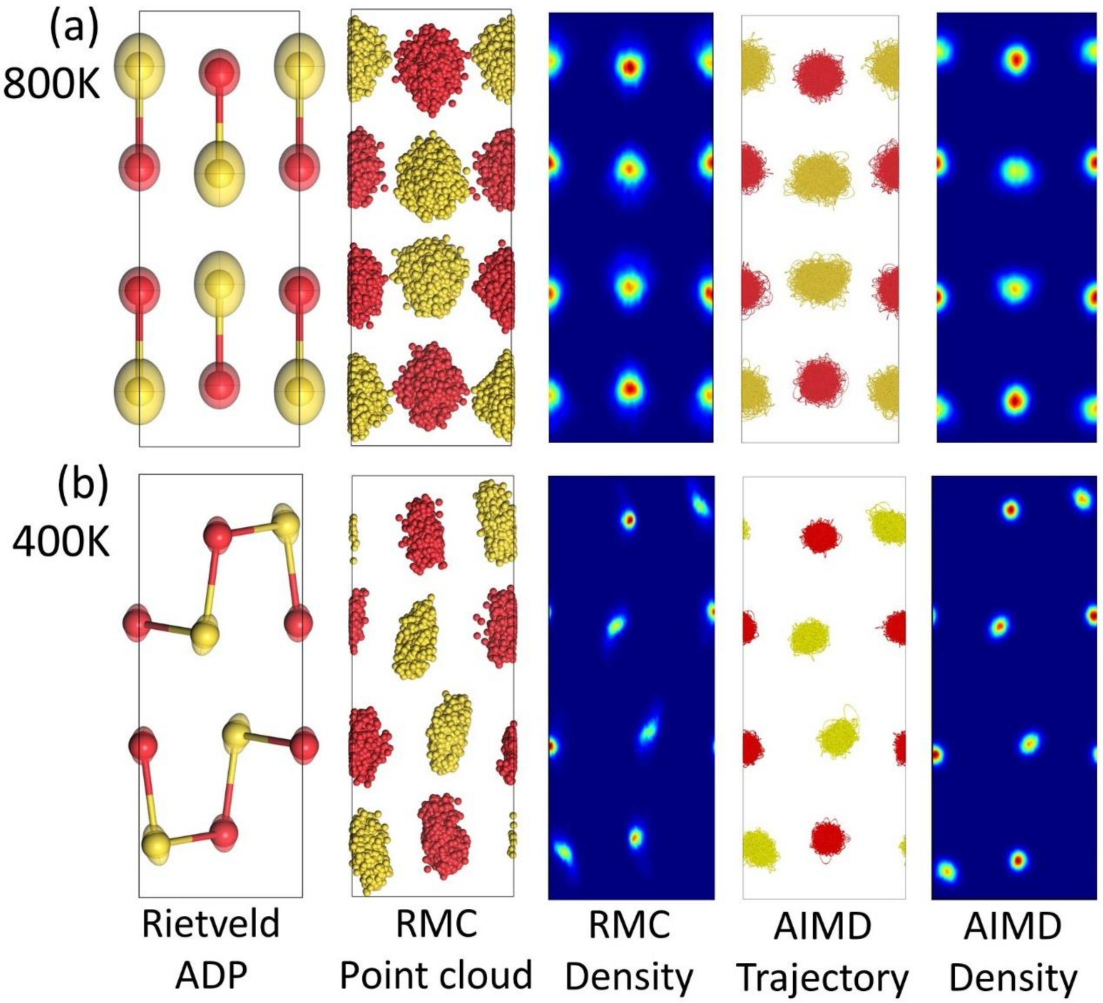

Fig. S12. Structure model results from Rietveld analysis, RMC analysis and AIMD simulation at (a) 800 K, (b) 400 K. Panels from left to right compare the unit cell contents corresponding to the determined ADP ellipsoids from Rietveld analysis (400 K using GeS-type (*Pnma*) model, 800 K using TlI-type (*Bbmm*, standard *Cmcm*) model, the atomic point cloud distributions (folded into a single unit cell) from RMC configurations, density plots of the RMC point clouds, AIMD trajectory projections (folded into a single unit cell), and corresponding density plots of the AIMD projections, respectively.

Supplementary Tables: Table S1. The agreement factor (R_w), lattice parameters and atomic position parameters from Rietveld refinement of neutron diffraction data using the GeS-type model (*Pnma*, with 43 parameters) and TlI-type model (*Bbmm*, with 39 parameters, standard *Cmcm*) space group model.

T(K)	R_w (%)	*Pnma* a (Å)	*Pnma* b (Å)	*Pnma* c (Å)	*Pnma* Sn x	*Pnma* Sn z	*Pnma* Se x	*Pnma* Se z
400	7.546	11.5216(15)	4.1623(5)	4.4282(5)	0.11856(16)	0.4008(3)	0.35442(16)	0.4807(3)
425	7.166	11.5420(15)	4.1729(5)	4.4182(5)	0.11898(17)	0.4040(4)	0.35464(18)	0.4805(3)
450	7.058	11.5454(15)	4.1747(5)	4.4170(5)	0.11906(17)	0.4044(4)	0.35474(18)	0.4806(3)
475	6.802	11.5543(14)	4.1795(4)	4.4139(5)	0.11915(16)	0.4056(3)	0.35477(14)	0.4806(2)
500	6.787	11.5565(14)	4.1810(4)	4.4120(5)	0.11919(17)	0.4062(4)	0.35482(17)	0.4807(3)
525	6.710	11.5685(14)	4.1875(4)	4.4071(5)	0.11931(18)	0.4083(4)	0.35489(17)	0.4807(3)
550	6.607	11.5789(14)	4.1937(4)	4.4009(5)	0.11958(19)	0.4106(4)	0.35505(17)	0.4809(3)
575	6.562	11.5915(14)	4.2008(4)	4.3950(5)	0.11983(20)	0.4129(4)	0.35528(18)	0.4813(3)
600	6.504	11.6054(14)	4.2083(4)	4.3888(5)	0.12035(21)	0.4155(4)	0.35582(19)	0.4816(3)
625	6.585	11.6130(15)	4.2146(4)	4.3807(5)	0.12056(22)	0.4183(4)	0.35604(19)	0.4818(3)
650	6.209	11.6305(15)	4.2245(4)	4.3753(5)	0.12115(22)	0.4219(4)	0.35645(20)	0.4822(4)
675	6.338	11.6373(15)	4.2326(5)	4.3655(5)	0.12170(22)	0.4264(5)	0.35656(19)	0.4825(4)
700	6.406	11.6471(16)	4.2426(5)	4.3550(6)	0.12227(22)	0.4317(5)	0.35680(19)	0.4830(4)
725	6.405	11.6575(16)	4.2546(5)	4.3434(6)	0.12278(21)	0.4385(6)	0.35705(19)	0.4834(5)
750	6.405	11.6725(16)	4.2712(5)	4.3310(6)	0.12379(23)	0.4473(7)	0.35772(21)	0.4850(6)
775	7.220	11.6898(16)	4.2896(6)	4.3124(7)	0.12530(29)	0.4602(9)	0.35905(23)	0.4952(7)
800	7.687	11.7102(17)	4.2974(7)	4.3121(7)	0.1267(3)	0.4726(12)	0.35994(26)	0.5012(10)
825	7.544	11.7212(17)	4.3013(7)	4.3175(7)	0.1265(3)	0.4723(12)	0.35979(27)	0.5012(10)
850	7.438	11.7173(17)	4.2995(7)	4.3162(7)	0.1268(3)	0.4733(12)	0.36014(27)	0.5007(10)
875	7.187	11.7234(18)	4.3013(7)	4.3188(7)	0.1269(3)	0.4732(12)	0.36017(27)	0.5012(10)
900	7.067	11.7332(18)	4.3048(7)	4.3235(7)	0.1269(3)	0.4729(12)	0.36008(27)	0.5013(10)
925	6.940	11.7379(18)	4.3061(7)	4.3260(7)	0.1270(4)	0.4729(12)	0.36014(28)	0.5020(10)
950	6.781	11.7400(19)	4.3064(7)	4.3279(7)	0.1269(4)	0.4730(12)	0.36012(28)	0.5014(11)
975	6.623	11.7530(19)	4.3104(8)	4.3331(7)	0.1273(4)	0.4731(12)	0.36020(29)	0.5023(11)
1000	6.460	11.7612(19)	4.3128(8)	4.3368(7)	0.1276(4)	0.4728(12)	0.36037(29)	0.5034(11)

T(K)	R_w (%)	*Cmcm* a (Å)	*Cmcm* b (Å)	*Cmcm* c (Å)	*Cmcm* Sn x	*Cmcm* Sn z	*Cmcm* Se x	*Cmcm* Se z
775	7.634	11.6953(18)	4.2898(6)	4.3130(7)	0.1278(3)	0.5	0.35951(27)	0.5
800	7.795	11.7134(17)	4.2983(8)	4.3116(8)	0.1277(3)	0.5	0.35958(27)	0.5
825	7.735	11.7252(18)	4.3022(8)	4.3174(8)	0.1275(3)	0.5	0.35940(29)	0.5
850	7.595	11.7221(19)	4.3005(8)	4.3166(8)	0.1279(4)	0.5	0.35985(30)	0.5
875	7.275	11.7277(18)	4.3023(8)	4.3187(8)	0.1280(4)	0.5	0.35986(28)	0.5
900	7.233	11.7374(19)	4.3060(8)	4.3233(8)	0.1281(4)	0.5	0.35981(28)	0.5
925	7.108	11.7424(19)	4.3073(8)	4.3259(8)	0.1282(4)	0.5	0.35996(29)	0.5
950	6.949	11.7446(19)	4.3076(8)	4.3277(8)	0.1281(4)	0.5	0.35986(29)	0.5
975	6.864	11.7571(20)	4.3116(8)	4.3329(8)	0.1287(4)	0.5	0.36014(30)	0.5
1000	6.618	11.7661(20)	4.3142(8)	4.3366(8)	0.1288(4)	0.5	0.36021(30)	0.5
